# Selective plasma exchange in ABO-incompatible kidney transplantation: comparison of substitution with albumin and partial substitution with fresh frozen plasma

**DOI:** 10.1038/s41598-020-58436-2

**Published:** 2020-01-29

**Authors:** Ako Hanaoka, Toshihide Naganuma, Daijiro Kabata, Yoshiaki Takemoto, Junji Uchida, Tatsuya Nakatani, Ayumi Shintani

**Affiliations:** 1 Department of Medical Devices, Osaka City University Hospital, Osaka, Japan; 2 Department of Urology, Osaka City University Graduate School of Medicine, Osaka, Japan; 3 Department of Medical Statistics, Osaka City University Graduate School of Medicine, Osaka, Japan

**Keywords:** Renal replacement therapy, Urology

## Abstract

We have performed selective plasma exchange (SePE) as apheresis before ABO-incompatible kidney transplantation since 2015. In this study, we divided the SePE sessions into two groups, those using albumin alone (Group A) and those partially using fresh frozen plasma (FFP) (Group F), and compared their clinical efficacies. A total of 58 sessions of SePE (Group A: n = 41, Group F: n = 17) were performed in 30 recipients of ABOi kidney transplantation during the study period and the decrease in isoagglutinin titers, changes in the levels of serum IgG and IgM as well as coagulation factors (fibrinogen, factor XIII), and incidence of side effects were retrospectively compared. There was a more significant decrease of isoagglutinin titers in Group F compared to Group A. Immunoglobulins and coagulants were replenished in Group F. Meanwhile, the incidence of side effects was significantly higher in Group F. SePE using FFP, which can effectively decrease isoagglutinins titers and replenish immunoglobulin and coagulation factors, may be a beneficial treatment modality as apheresis before ABO-incompatible kidney transplantation, in spite of a disadvantage that there are many side effects.

## Introduction

Desensitization protocol for ABO-incompatible (ABOi) kidney transplantation, antibody removal therapy by pre-transplant apheresis to lower the isoagglutinin titers is gradually less common for patients with low antibody titers at baseline and those who are relieved by immunosuppressive therapy in some institutions^1–6^. However, most institutions perform apheresis several times before kidney transplantation^7–12^.

As the modality for apheresis, double-filtration plasmapheresis (DFPP), simple plasma exchange (PE), or immunoadsorption (IA) is used^9,13–15^. DFPP and PE using albumin as substitution fluid is known to increase risks for peri-operative bleeding due to decreased coagulation factor^9,16–18^. PE using fresh frozen plasma (FFP) causes FFP-induced allergic reactions^19,20^. IA is considered to be a very effective method, as GLYCOSORB-ABO columns that specifically absorb anti-blood type antibodies are used with minimal side effects and loss of coagulation factors. However, on the other hand, there have been reports that bleeding complications occur more frequently compared to ABO-compatible renal transplantation^21^. Moreover, the problem of IA is that columns to be used are very expensive (about 3,000 Euros^14^) and is not currently applied and unavailable in Japan.

Selective plasma exchange (SePE) is a type of PE which uses a membrane plasma separator with a smaller pore size (EVACURE PLUS EC-4A10, KAWASUMI Laboratories, Inc., Tokyo, Japan; sieving coefficients: albumin; 0.61, IgG; 0.44, IgM; 0, fibrinogen; 0) compared to conventional membrane plasma separators^22^. A major feature of SePE is that small and medium molecular weight substances are removed, while larger molecular weight substances are not (e.g. coagulation factors). Recently, SePE using albumin has become increasingly noted, because of its fewer side effects compared to PE using FFP, and its economic merits, as albumin is cheaper than FFP (SePE using albumin is half the price of PE using FFP), as well as because there is fewer loss of coagulation factors compared to DFPP and PE using albumin. SePE is being performed more and more in cases where the target substance can be removed by SePE^23–26^.

Since 2015, we have performed SePE as pretransplant apheresis in ABOi kidney transplantation with favorable results^27^. Isoagglutinin titers have been satisfactorily controlled by SePE alone in patients with low isoagglutinin titers and by SePE in combination with PE and DFPP in patients with high isoagglutinin titers without incidence of antibody-mediated rejection (AMR)^27^. However, because the amount of coagulation factors tends to be low in patients with already low levels at baseline and in patients receiving multiple DFPP sessions^18,28^, we partly use FFP as the substitution fluid in order to reduce peri-operative bleeding risks. In this study, we divided all the cases of SePE into two groups, substitution with albumin and partial substitution with FFP, and the clinical results were compared.

## Materials and Methods

### Study design and participants

A retrospective case-series study was conducted on patients who underwent ABOi kidney transplantation at the Department of Urology, Osaka City University Hospital from January 2015 to December 2018. All donor kidneys were from living relatives. The inclusion criteria were ABO-i KTRs who received at least one session of SePE. Anti-HLA antibody-positive patients were excluded, and a total of 30 KTRs were enrolled in this study. This study participant includes 15 KTRs enrolled in our previous study^27^. A total of 58 SePE sessions were performed as a method to remove antibodies before transplantation, which were divided into two groups according to the substitution fluid used: substitution with albumin (Group A; n = 41) and partial substitution with FFP (Group F; n = 17). The decrease in isoagglutinin titers, changes in serum IgG and IgM levels, changes in coagulation factors (fibrinogen, factor XIII), and incidence of side effects were studied retrospectively and compared between the two groups. The study protocol was conducted in accordance with the Declaration of Helsinki and Declaration of Istanbul on organ trafficking and transplant tourism and approved by the ethics committee of Osaka City University Graduate School of Medicine (No. 4009). Informed consent was obtained in the form of opt-out; i.e., we provided the patients with information explaining the proposed research project (the purpose, required individual data and duration of the study) by means of an information sheet or hospital website and gave them the opportunity to opt out. The patient background is given in Table 1.

**Table 1:** Contents of Antibody Removal Therapy including Selective Plasma Exchange.

Case	Order and Frequency of Apheresis Therapy
1	DFPP × 1 ⇒ SePE(A) × 6
2	SePE(A) × 2 ⇒ PE × 6
3	DFPP × 1 ⇒ PE × 1 ⇒ SePE(A) × 1
4	DFPP × 1 ⇒ SePE(A) × 1
5	DFPP × 1 ⇒ PE × 1 ⇒ DFPP × 1 ⇒ SePE(A) × 1
6	DFPP × 2 ⇒ SePE(A) × 1
7	DFPP × 2 ⇒ SePE(A) × 1
8	DFPP × 2 ⇒ SePE(F) × 1
9	DFPP × 2 ⇒ SePE(F) × 1
10	DFPP × 2 ⇒ SePE(F) × 1
11	SePE(F) × 1 ⇒ DFPP × 1 ⇒ SePE(F) × 1 ⇒ DFPP × 1 ⇒ SePE(A) × 1
12	DFPP × 1 ⇒ SePE(F) × 1
13	DFPP × 1 ⇒ SePE(F) × 1
14	SePE(A) × 1 ⇒ SePE(F) × 1
15	DFPP × 1 ⇒ SePE(F) × 2
16	SePE(A) × 2
17	SePE(A) × 2
18	SePE(A) × 2
19	SePE(A) × 2
20	SePE(A) × 2
21	PE × 1 ⇒ SePE(A) × 3
22	SePE(F) × 2
23	SePE(A) × 2
24	SePE(A) × 1
25	SePE(A) × 2
26	SePE(A) × 1
27	SePE(A) × 2
28	SePE(A) × 3
29	SePE(A) × 1 ⇒ PE × 1 ⇒ SePE(A) × 1 ⇒ SePE(F) × 1
30	SePE(F) × 1 ⇒ SePE(A) × 1 ⇒ SePE(F) × 2

SePE (A), selective plasma exchange using 5% albumin solution as the substitution fluid; SePE (F), selective plasma exchange partially using fresh frozen plasma as the substitution fluid; DFPP, double-filtration plasmapheresis; PE, simple plasma exchange.

### Desensitization protocols

Desensitization was conducted by immunosuppressive therapy centering on rituximab and antibody removal by pretransplant apheresis to lower the isoagglutinin titers (immunoglobulin G (IgG) and M (IgM)) to ≤ 1:16^8,17,29,30^.

1. Immunosuppression protocol: Immunosuppression protocol was derived from our previous studies^4,31^. The details of the protocol are shown in the Supplemental Material.
2. Apheresis protocol: For removal of isoagglutinins, the patients received at least 1 session of apheresis including SePE before transplantation until the isoagglutinins decreased to ≤1:16. The number of sessions was determined according to the level of isoagglutinin titers. In principle, in patients with high titers (>1:64) at baseline, SePE was performed after the isoagglutinin titers were reduced to some extent by DFPP or PE. In patients with low baseline fibrinogen levels and patients whose fibrinogen levels decreased by repeated DFPP sessions, SePE using partial substitution with FFP was performed instead of SePE using substitution with albumin. In patients with low fibrinogen levels after apheresis, FFP was transfused so that pretransplant fibrinogen levels became >200 mg/dl.

### Conditions for performing SePE

**Figure 1:**
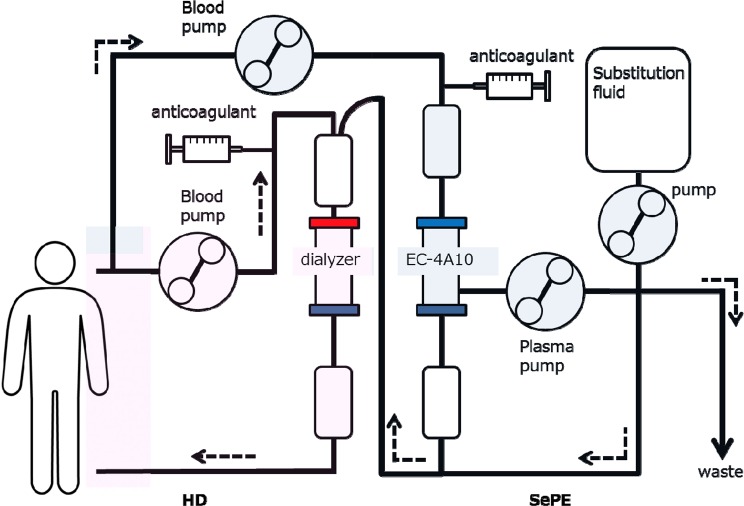
Selective plasma exchange circuit with hemodialysis^27^.

Conditions for performing SePE were derived from our previous studies^27^. The SePE circuit was showed in the Fig. 1^27^. The details of conditions are shown in the Supplemental Material. In brief, SePE was performed using a KM-9000 (SANYO ELECTRONIC INDUSTRIES Co., Ltd. Okayama, Japan), TR55X (TORAY MEDICAL Co., Ltd. Tokyo, Japan) or ACH-Σ (PLASAUTO Σ in overseas models) (ASAHIKASEI MEDICAL Co., Ltd. Tokyo, Japan) blood purification system and an EVACURE PLUS EC-4A10 (EVACLIO, EC-4C in overseas models) (KAWASUMI LABORATORIES, Inc., Tokyo, Japan) selective plasma separator^27^. During SePE, blood flow was maintained at 100 ml/min with a plasma separation rate of 30 ml/min, and unfractionated heparin or nafamostat mesilate was used as the anticoagulant. In tandem HD and SePE, SePE was performed in parallel with the HD circuit, with a blood flow rate of 100 mL/min into the SePE circuit.

**Figure 2:**
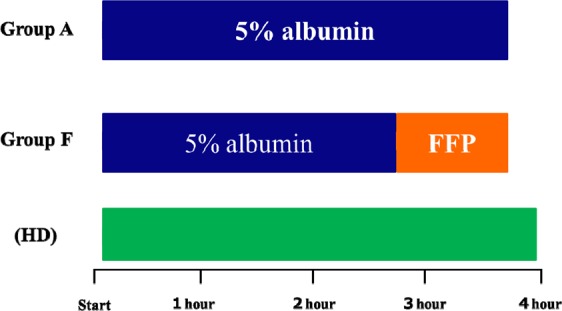
Time schedule of substitution fluid administration.

The target processed PV of substitution fluid was set at 2PV. As the substitution fluid, 5% albumin only was used in Group A, while in Group F, 5% albumin was used at 3/4 of the total volume followed by FFP at 1/4 of the total volume (Fig. 2). Basically, SePE is completed within the 4-hour HD time in both Group A and F, but when the total volume of the replacement fluid is large, the HD time is extended. As for medical costs, it was 30% higher in Group F compared to Group A.

### Measurement of clinical data

Methods of measurement of clinical data were derived from our previous studies^27^.

At the tandem HD and SePE therapy, preoperative blood sampling was performed at the beginning of HD and postoperative one at the end of HD (SePE was completed before sampling in all cases).

The details of methods are shown in the Supplemental Material.

### Statistical analysis

Baseline characteristics were summarized using medians and inter quantile ranges for continuous variables and percentages and counts for categorical variables. And differences of these variables between the patients contained within group A and F. To examine whether following serum value changes over time, we used multivariable linear regression models including the isoagglutinin titers (IgG, IgM), fibrinogen and factor XIII levels as a dependent variable separately and a cross product term between the group variable and the variable indicating examination time within each session as an independent variable. In these regression models, analyzes were performed with adjustment for the use of anticoagulants and tandem HD. And all dependent variables were used with normal logarithmic transformation to satisfy the assumption of the error distribution normality of a linear regression model. Furthermore, we compared the incidence proportion of the side-effects occurred within each session between A and F groups using a multivariable logistic regression model with the variable indicating occurrence of the side effects as the function of the group variable with adjustment for the use of anticoagulants and tandem HD. In all regression models, Huber-White robust sandwich estimator of variance covariance matrix was considered to account for dependence in repeated measures because the dataset contained the repeated measures within a patient.

All statistical analyzes were conducted with two-sided significance level of 5% using R software version 3.5.0 (https://www.r-project.org/foundation/) with “rms” and “RcmdrPlugin.EZR” packages.

## Results

### Contents of antibody removal therapy including SePE

A total of 58 SePE sessions were performed in 30 recipients of ABOi kidney transplantation (Group A: n = 41, Group F: n = 17) (Table 1). In 14 recipients, SePE alone was performed, while in 16 patients, SePE was performed in combination with DFPP and PE.

### Characteristics of the study subjects

In 21 patients, SePE was performed in tandem with HD, and SePE alone was performed in 9 patients (5 peritoneal dialysis patients, 4 preemptive kidney transplantation patients) (Table 2). Median mismatch (antigen) of human leukocyte antigen (HLA) was 3 [3, 4]. Median dialysis duration was 20.5 [5.3, 51.8] months. Prevalence of diabetes mellitus (DM) was 14 (46.7%) recipients.

**Table 2:** Characteristics of the Study Subjects.

Case	Pre-transplant renal replacement therapy	SePE + HD	SEX/Age	Donor (Sex/Age)	Relationship	Blood type	HLA mismatch (antigen)	Dialysis duration (months)	DM	Anti-A/B IgG titers	Anti-A/B IgM titers
										Base	Base
1	PEKT	NO	M/67	F/66	Spouse	AB + ⇒ O+	4	0	0	256/256	16/16
2	HD	YES	F/60	M/58	Spouse	B+ ⇒ O+	5	131	0	1024	512
3	HD	YES	F/64	M/59	Spouse	A+ ⇒ B+	4	130	1	16	16
4	HD	YES	F/32	M/55	Parent	AB+ ⇒ B+	1	4	0	8	8
5	HD	YES	F/39	M/61	Parent	B+ ⇒ O+	3	9	0	256	32
6	PD	NO	M/44	F/67	Parent	B+ ⇒ O+	3	40	0	64	32
7	PD	NO	F/69	M/69	Spouse	AB+ ⇒ B+	3	10	1	16	16
8	HD	YES	F/68	M/69	Spouse	B+ ⇒ A+	4	99	0	32	32
9	HD	YES	F/34	M/58	Parent	B+ ⇒ A+	2	23	1	8	8
10	HD	YES	M/66	M/65	Spouse	B+ ⇒ A+	6	59	0	64	16
11	HD	YES	M/65	M/53	Spouse	AB+ ⇒ A+	4	55	1	32	128
12	HD	YES	M/63	M/31	Parent	AB+ ⇒ A+	2	71	1	4	8
13	HD	YES	M/52	F/49	Spouse	B+ ⇒ A+	4	24	1	16	16
14	PEKT	NO	M/70	M/72	Spouse	A+ ⇒ B+	5	0	0	16	16
15	HD	YES	M/66	F/73	Spouse	B+ ⇒ A+	3	1	1	256	256
16	HD	YES	F/44	F/48	Sibling	B+ ⇒ A+	3	10	0	1	2
17	HD	YES	M/52	F/50	Spouse	B+ ⇒ A+	5	16	1	4	4
18	HD	YES	M/53	F/80	Spouse	B+ ⇒ A+	5	1	1	8	4
19	HD	YES	M/53	F/50	Spouse	B+ ⇒ A+	2	9	0	8	4
20	HD	YES	M/53	F/55	Spouse	B+ ⇒ A+	3	27	0	2	2
21	PD	NO	M/51	F/50	Spouse	A+ ⇒ B+	4	135	1	32	32
22	HD	YES	M/47	F/73	Parent	A+ ⇒ B+	3	18	0	16	8
23	PEKT	NO	M/37	M/39	Spouse	AB+ ⇒ B+	2	0	1	16	8
24	PD	NO	M/53	M/55	Sibling	B+ ⇒ O+	0	42	1	16	16
25	HD	YES	M/31	M/57	Parent	AB+ ⇒ B+	3	36	1	16	16
26	PEKT	NO	M/49	F/42	Spouse	AB+ ⇒ B+	5	0	1	2	1
27	HD	YES	M/65	F/54	Spouse	AB+ ⇒ A+	3	27	0	8	16
28	PD	NO	M/62	F/60	Spouse	B+ ⇒ A+	3	65	0	32	32
29	PEKT	YES	M/25	M/53	Parent	A+ ⇒ O+	3	0	0	64	32
30	HD	YES	M/54	F/50	Spouse	B+ ⇒ 0+	4	14	0	256	16

SePE, selective plasma exchange; HD, hemodialysis; PD, peritoneal dialysis; PEKT, preemptive kidney transplantation; M, male; F, female; HLA, human leukocyte antigen; DM, diabetes mellitus; IgG, immunoglobulin G; IgM, immunoglobulin M.

### Results of the study subjects

Median total volume of substitution fluid (mL) at SePE was 6000 [4500, 6000] mL (Table 3). Median FFP substitution fluid (mL) at SePE was 1000 [1000, 1275] mL. After their transplants, all patients have made satisfactory progress without incident of AMR. Prevalence of acute cellular rejection (AR) was 5 (16.7%) recipients. Four of them was diagnosed with Banff IA and one (Case 6) was diagnosed with Banff IB. Median estimated glomerular filtration rate (eGFR) at discharge was 39.36 [34.02, 52.60] mL/min/1.73 m^2^. With regard to complication at perioperative period, postoperative bleeding was found in cases 2 and 15.

**Table 3:** Results of the Study Subjects.

Case	total volume of substitution fluid (ml) at SePE	AMR	AR	eGFR at discharge (mL/min/1.73 m^2^)	Complication at perioperative period	Anti-A/B IgG titers			Anti-A/B IgM titers		
						at Tx	at POD1	Max	at Tx	at POD1	Max
1	5500	0	0	38.65	Pyelonephritis, CMV antigenemia (+)	32/128	32/32	256/256	8/8	4/4	16/16
2	3000	0	0	28.62	Postoperative bleeding	64	32	1024	1	1	1024
3	7000	0	0	45.97	CMV antigenemia (+)	8	16	32	2	2	16
4	4000	0	0	40.47		1	<1	16	1	<1	16
5	7000	0	0	56.91		4	2	256	2	1	32
6	7000	0	1	31.02		8	4	64	2	1	32
7	4500	0	1	34.52	CMV antigenemia (+)	<1	<1	16	1	1	16
8	3750	0	0	37.36	Lymphocele	2	2	32	2	1	32
9	3750	0	0	42.3	Lymphocele	1	<1	64	<1	1	32
10	5000	0	0	38.53		2	2	64	1	1	16
11	6000	0	1	34.77	Herpes zoster	4	2	128	2	2	128
12	4500	0	0	32.79		1	<1	16	1	2	16
13	4500	0	1	26.62	Lymphocele	2	<1	16	1	1	16
14	5000	0	0	48.11	Lymphocele	4	<1	16	4	<1	16
15	3500	0	0	30.27	Postoperative bleeding	2	2	256	4	2	256
16	4000	0	0	61.77		<1	<1	1	1	1	2
17	7000	0	1	40.06		1	<1	4	1	1	4
18	6000	0	0	36.87	Lymphocele	2	2	16	2	1	8
19	6000	0	0	54.31		2	<1	8	4	2	4
20	6000	0	0	37.38		1	<1	1	1	<1	2
21	6000	0	0	50.95		1	<1	32	1	1	32
22	6000	0	0	31.84		1	<1	16	2	2	8
23	5500	0	0	67.69		8	2	32	4	2	32
24	7000	0	0	58.24	Lymphocele	8	<1	16	8	<1	16
25	7000	0	0	51.72		2	1	16	2	2	16
26	7000	0	0	57.2		<1	2	2	<1	<1	1
27	6000	0	0	33.85		8	1	8	8	4	16
28	6000	0	0	68.25	Pyelonephritis	4	<1	32	4	1	32
29	6000	0	0	52.89		16	16	128	2	1	64
30	5500	0	0	28.15		32	16	256	8	2	16

SePE, selective plasma exchangeusing; AMR, antibody-mediated rejection; AR, Acute cellular rejection; eGRF,estimated glomerular filtration rate; CMV, cytomegalovirus; IgG, immunoglobulin G; IgM, immunoglobulin M; Tx, kidney transplantation; POD1, postoperative day1.

Except for Patient 1, 2, and 30, whose baseline isoagglutinin titers were high, isoagglutinin titers were well controlled (≤1:16 at transplant). In Patient 1 and 2, isoagglutinin titers did not decrease enough, so splenectomy was also performed. In Patient 30, IgG isoagglutinin titers only decreased to 1:32, but the attending surgeon decided to go ahead with the transplantation.

### Comparison of decrease in anti-blood type antibody titers (Figs. 3, 4)

**Figure 3:**
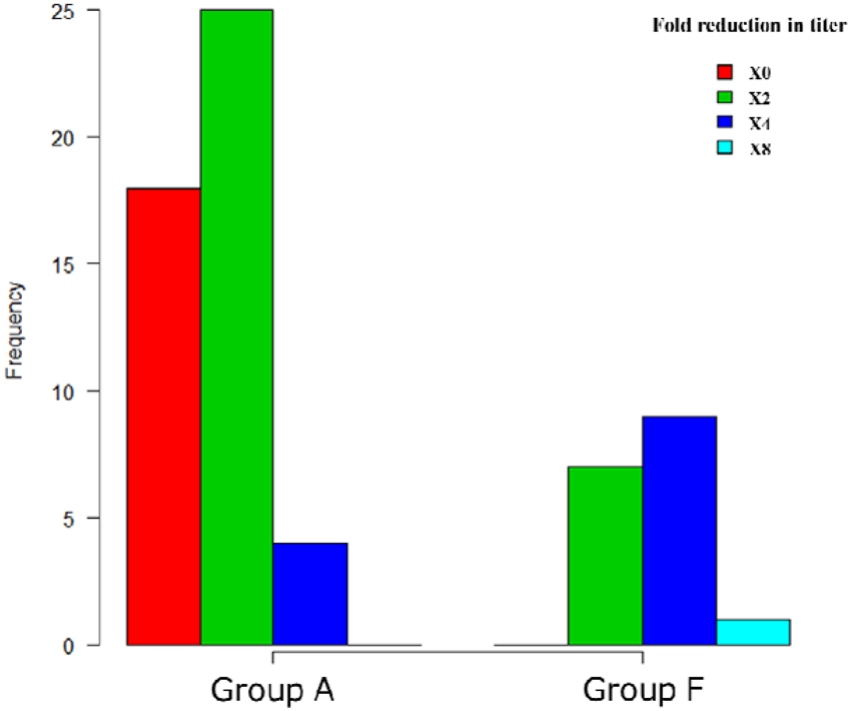
Distribution of decrease in IgG antibody titers.

**Figure 4:**
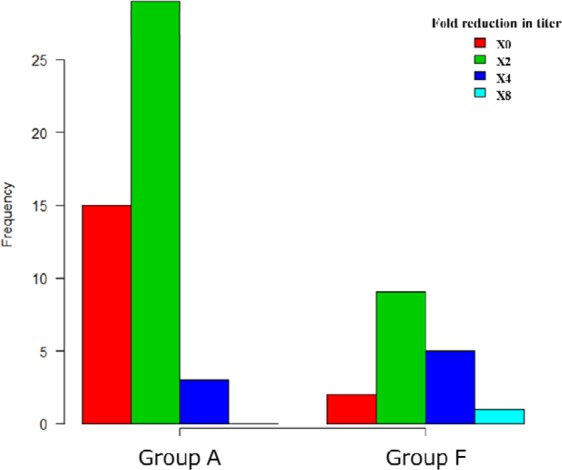
Distribution of decrease in IgM antibody titers.

The median decrease in IgG isoagglutinin titer was by 2 [0, 1] fold in Group A and 4 [1, 2] fold in Group F, and there was a more significant decrease in Group F (p < 0.0001, Fig. 3). The median decrease in IgM isoagglutinin titer was by 2 [0, 1] fold in Group A and 2 [1, 2] fold in Group F, and there was a more significant decrease in Group F (p = 0.0044, Fig. 4). In more than 30% of the sessions in Group A, both IgG and IgM isoagglutinin titers did not decrease at all.

### Comparison of changes in serum IgG and IgM (Figs. 5, 6)

**Figure 5:**
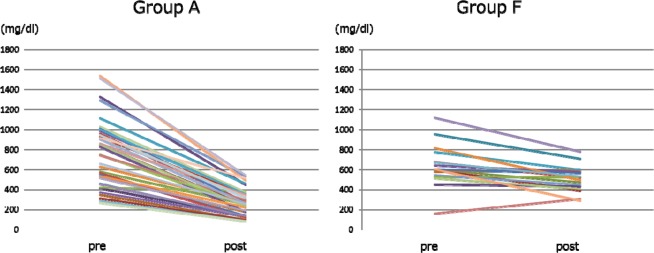
Changes in serum IgG.

**Figure 6:**
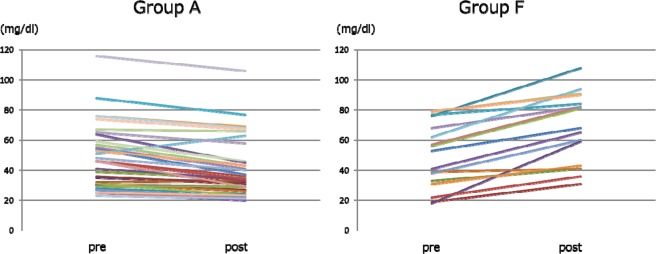
Changes in serum IgM.

The changes in serum IgG and IgM after SePE in Group A and F are shown in Figs. 5 and 6. The median serum IgG levels before and after SePE was 787.0 [524.5, 949.5] and 255.5 [146.0, 353.8] mg/dl in Group A, and 598.0 [545.0, 673.0] and 520.0 [434.00, 574.0] mg/dl in Group F. There was a significant difference in the amount of change between the two groups (p < 0.0001, Fig. 5). The median serum IgM levels before and after SePE was 46.0 [30.5, 64.5] and 34.5 [27.5, 55.0] mg/dl in Group A and 53.0 [33.0, 68.0] and 68.0 [43.0, 84.0] mg/dl in Group F. There was a significant difference in the amount of change between the two groups (p < 0.0001, Fig. 6).

### Comparison of changes in coagulation factors (Figs. 7, 8)

**Figure 7:**
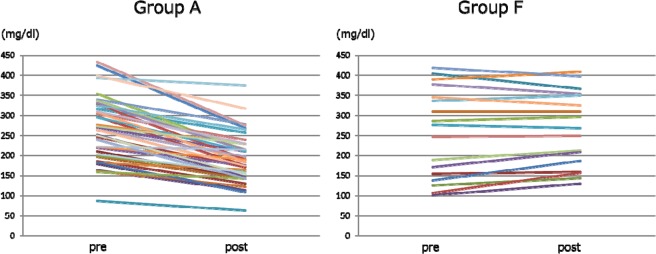
Changes in fibrinogen.

**Figure 8:**
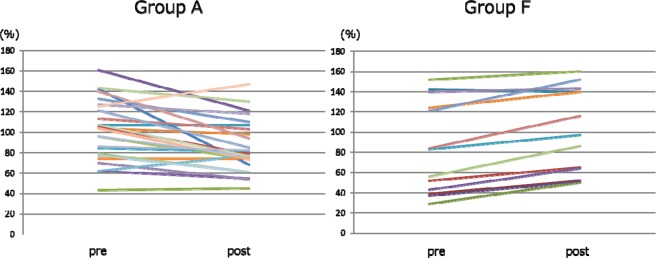
Changes in factor XIII.

The changes in serum fibrinogen levels after SePE in Group A and F are shown in Fig. 7. The median fibrinogen levels before and after SePE was 264.0 [201.8, 318.0] and 186.5 [145.8, 229.0] mg/dl in Group A and 276.0 [155.0, 345.0] and 268.0 [187.0, 350.0] mg/dl in Group F, and there was a significant difference in the amount of change between the two groups (p < 0.0001). The changes in factor XIII levels after SePE in Group A and F are shown in Fig. 8. The median factor XIII levels were 104.5 [80.3, 126.5] and 79.0 [73.0, 103.0] % in Group A and 83.0 [43.0, 124.0] and 97.0 [64.0, 140.0] % in Group F, and there was a significant difference in the amount of change between the two groups (p < 0.0001).

### Comparison of incidence of side effects (Fig. 9, Table 4)

**Figure 9:**
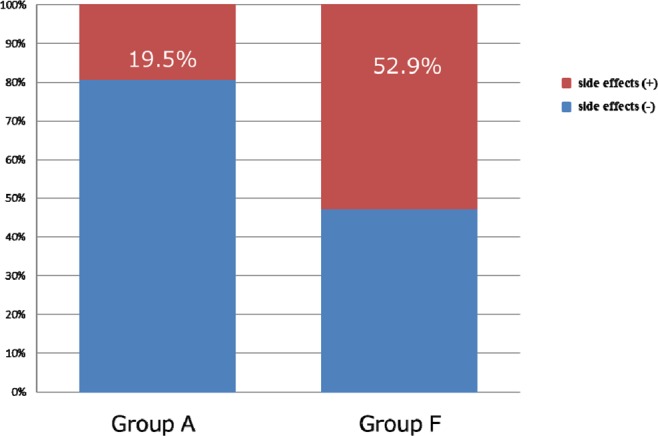
Comparison of incidence of side effects.

Side effects occurred in 17 out of the 58 sessions, and the incidence was significantly higher in Group F (8/17) compared to group A (9/41) (p = 0.0182, Fig. 9). The side effects experienced are listed in Table 4. All side effects were successfully treated and did not lead to suspension of treatment.

**Table 4:** Contents of side effects.

	Side effects	Number
Group F	rashes	6
	nausea	2
	numbness	1
Group A	numbness	2
	leg cramps	2
	poor feeling, hot flashes	1
	nausea, decrease of blood pressure	1
	abdominal pain	1
	epigastric discomfort	1

## Discussion

A total of 58 sessions of SePE (Group A: n = 41, Group F: n = 17) were performed in 30 recipients of ABOi kidney transplantation during the study period. Isoagglutinin titers were favorably controlled (≤1:16 at transplantation) in 27 of the recipients, except for 3 recipients with high titers, and none of the recipients experienced AMR. There was a more significant decrease of isoagglutinin titers in Group F compared to Group A. Immunoglobulins and coagulants were replenished in Group F, because FFP was used as the substitution fluid. Meanwhile, the incidence of side effects was significantly higher in Group F. This is the first report comparing substitution with albumin and partial substitution with FFP in SePE as a method to remove isoagglutinins in ABOi kidney transplantation.

In order to inhibit the incidence of AMR after kidney transplantation, pre-transplant apheresis to remove isoagglutinins is performed, but isoagglutinin titers did not decrease at all in more than 30% of Group A (Figs. 3 and 4). In group F, IgG isoagglutinin titers decreased in all of the sessions, and there was a more significant decrease in Group F compared to Group A (Fig. 3). These results suggested that the use of FFP as substitution fluid in SePE was effective in decreasing IgG isoagglutinin titers. There may be influences of IgG contained in FFP such as competitive inhibition, but the detailed mechanism is not clear and further investigation is needed. In both groups, SePE was able to decrease IgM isoagglutinin titers to some extent (Fig. 4). However, this decrease in IgM was not comparable to the decrease in IgG. Therefore, PE or DFPP should be performed first in patients with high IgM isoagglutinin titers. Because IgM is mainly found in the blood unlike IgG, once IgM titers are reduced by PE or DFPP as the initial treatment, these patients can be treated by using SePE thereafter.

To maintain serum IgG levels >500 mg/ml has been reported to be effective to avoid bacterial infection^32,33^. However, serum IgG levels tend to decrease in DFPP and SePE using substitution with albumin. In this study, the median serum IgG level after SePE was 255.5 mg/ml in Group A and lower compared to Group F (Fig. 5). In Group F, the median serum IgG level was maintained at 520.0 ml/dl, because IgG is contained in FFP. These results indicated that substitution with FFP may be effective in preventing bacterial infection.

When SePE using albumin is performed, approximately 20 to 40% of coagulation factors are removed^27,34,35^. Although the sieving coefficient of fibrinogen was 0 and that of factor XIII was 0.17 according to the manufacturer’s reported value of EC-4A10, fibrinogen was decreased by 29.5% and factor XIII by 24.4% in Group A, and replenishment of FFP was necessary. Okubo *et al*.^36^ reported that this was caused by the adhesion of fibrinogen fiber-like substances to the membrane by electron microscopic observation of EC-4A10. Because coagulation factors decrease in patients with low baseline coagulation factor levels and patients receiving multiple DFPP sessions^18,28^, it is not desirable to perform SePE using albumin in these patients from the viewpoint of maintaining coagulation factors. In Group F, because coagulation factors are contained in FFP, it was possible to maintain normal coagulation factor levels (Figs. 7 and 8). Actual volume of FFP substitution fluid in Group F ranged from 1000 to 1500 mL. The pore size of column used in SePE is smaller than that in simple PE and few coagulation factors pass through. Therefore, the advantage of SePE using FFP as substitution fluid is to supplement sufficient coagulation factors more than simple SE at the same volume. These results suggested that SePE using partial substitution with FFP may be effective in preventing peri-operative bleeding. However, a well-designed clinical study must be conducted in future to verify the efficacy of this treatment method.

When FFP is used in PE, side effects such as allergic reactions become an issue, and in some cases, PE itself or HD performed in tandem with PE needs to be suspended^19,20^. In group A, side effects related to FFP was avoided. However, the incidence of side effects was higher in Group F compared to Group A, and these side effects occurred soon after switching to FFP. KTRs usually undergo HD, and the time schedule for patients receiving SePE in tandem with HD is shown in Fig. 2. Because about 3 hours of HD is completed when switching to FFP, the advantage with this system is that even if patients cannot continue treatment due to side effects, they have already completed HD. All side effects were treatable and did not lead to suspension of treatment.

There are several limitations in this study. Firstly, because the sample size of 30 patients was small, further studies with a larger sample size are necessary to confirm our results. Secondly, long-term effects on graft function need to be studied. Thirdly, we used one fourth of the amount of albumin for FFP, but the adequate amount of FFP must also be investigated. Fourthly, the present study was only on SePE patients, but comparisons with conventional methods (PE, DFPP, IA) need to be done.

In conclusion, our results suggested that the use of FFP in SePE was effective in decreasing isoagglutinin titers. Because there were cases in which isoagglutinin titers did not decrease at all by using albumin alone, SePE using FFP, which can effectively decrease isoagglutinins titers and replenish immunoglobulin and coagulation factors, may be a beneficial treatment modality as apheresis before ABO-incompatible kidney transplantation, in spite of a disadvantage that there are many side effects.

## Supplementary information

Supplemental material.
