# Supplementary material for: Selective plasma exchange in ABO-incompatible kidney transplantation: comparison of substitution with albumin and partial substitution with fresh frozen plasma
Source: Sci Rep. 2020 Jan 29;10:1434. doi: 10.1038/s41598-020-58436-2 (PMC6989510; doi:10.1038/s41598-020-58436-2)
Supplement: Supplementary file 1 — Supplemental material. [file 41598_2020_58436_MOESM1_ESM.pdf]

Title: Selective plasma exchange in ABO-incompatible kidney transplantation: comparison of substitution with albumin and partial substitution with fresh frozen plasma

Author's full names:

Ako Hanaoka, CE[1)], Toshihide Naganuma, MD, PhD*[2)], Daijiro Kabata, MPH[3)], Yoshiaki Takemoto, MD, PhD[2)], Junji Uchida, MD, PhD[2)], Tatsuya Nakatani, MD, PhD[2)] and Ayumi Shintani, MPH, PhD[3)]

Institutions:

1. Department of Medical Devices, Osaka City University Hospital, Osaka, Japan
2. Department of Urology, Osaka City University Graduate School of Medicine, Osaka, Japan
3. Department of Medical Statistics, Osaka City University Graduate School of Medicine, Osaka, Japan

Supplemental material

Immunosuppression protocol

Mycophenolate mofetil (MMF) 1 g/day (0.5 g/day for patients aged 65 years and older) was started 4 weeks before transplantation. All patients with titers less than 1:512 underwent administration of a single dose of rituximab (150 mg/m^2) at 2 weeks before transplantation. The patients with titers 1:512 and over and those with rebound of titers received rituximab (150 mg/m^2) at 2 weeks before and on the day of transplantation. Posttransplant immunosuppression consisted of calcineurin inhibitor (tacrolimus or cyclosporine initiated 3 days prior to transplantation), MMF or everolimus, steroid, and basiliximab.

Conditions for performing SePE

Apheresis was performed using KM-9000 (SANYO ELECTRONIC INDUSTRIES Co., Ltd. Okayama, Japan) or TR55X (TORAY MEDICAL Co., Ltd. Tokyo, Japan) or ACH-Σ (PLASAUTO Σ in overseas models) (ASAHIKASEI MEDICAL Co., Ltd. Tokyo, Japan) blood purification system. SePE was performed using the EVACURE PLUS EC-4A10 (KAWASUMI LABORATORIES Inc., Tokyo, Japan; sieving coefficients: albumin; 0.61, IgG; 0.44, fibrinogen; 0) as the plasma separator. During SePE, blood flow was maintained at 100 ml/min with a plasma separation rate of 30

ml/min, and unfractionated heparin or nafamostat mesilate was used as the anticoagulant. Plasma volume (PV) was calculated using the following equation: PV= (BW/13) x (100-Ht)/100, where BW and Ht indicate body weight (kg) and hematocrit (%), respectively. The target processed PV was set at 2PV. In tandem HD and SePE, SePE was performed in parallel with the HD circuit, with a blood flow rate of 100 mL/min into the SePE circuit. In order to prevent the decrease in blood pressure due to the difference in colloid osmotic pressure between the plasma and substitution fluid, a clit-line monitor was connected to the blood circuit, and intravascular blood volume was monitored.

Measurement of clinical data

Isoagglutinin titers were measured by the tube centrifugation test, anti-A/B IgM titers were determined using the saline agglutination technique, and IgG titers were evaluated using the indirect Coombs' test. Changes in isoagglutinin titers and serum IgG, IgM, fibrinogen and factor XIII levels were studied, in addition to any adverse effects.
